# Complete Genome Sequences for Two *Myoviridae* Strains Infecting Cyanobacteria in a Subarctic Lake

**DOI:** 10.1128/MRA.01339-19

**Published:** 2020-03-05

**Authors:** Alice V. Lévesque, Mary Thaler, Simon J. Labrie, Catherine Marois, Antony T. Vincent, Anne-Marie Lapointe, Alexander Culley

**Affiliations:** a Department of Biochemistry, Microbiology, and Bioinformatics, Université Laval, Québec, Québec, Canada; KU Leuven

## Abstract

We isolated two closely related strains that belong to the *Myoviridae* family and infect cyanobacteria in a shallow subarctic rock basin lake. Their host was identified as a member of the *Synechococcus-Cyanobium* complex. Sequenced genomes of the two phages were 244,930 bp and 243,633 bp. We describe their annotation and highlight some noteworthy features.

## ANNOUNCEMENT

Cyanobacteria often dominate picophytoplankton biovolume in high-latitude freshwater systems (1). The genomes of phages infecting these cyanobacteria are expected to give insight into top-down control of this group.

**FIG 1:**
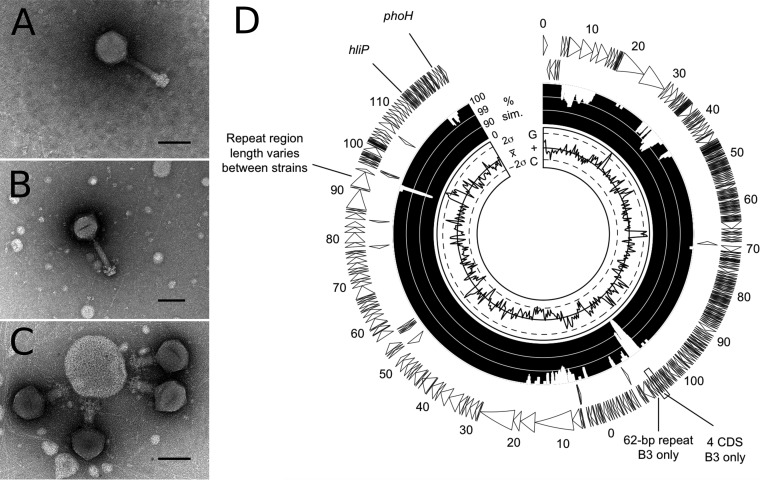
(A to C) Transmission electron micrographs of cyanophages B3 (A), B23 (B), and B23 in its contracted form (C). Scale bars, 100 nm. (D) Circular annotated genome of strain B3, showing differences from B23. Putative CDSs identified using RAST are shown as arrows for the positive and negative strands. Percent sequence identity between B3 and B23 (shown on a logarithmic scale) and G+C content (relative to the genome-wide mean of 34.8%) are shown on the inner rings. Selected features discussed in the text are indicated.

We isolated cyanophage strains by using a viral concentrate obtained by collecting water from the surface and bottom of a small rock basin lake in Nunavik, Canada (55°16′N, 77°44′W) and prefiltering it sequentially at 20, 0.45, and 0.22 μm. This concentrate was used to inoculate a mixture of 24 cultures from the Polar Cyanobacteria Culture Collection at the Centre for Northern Studies (Université Laval, Québec, Canada). A process of filtration at 0.45 μm to obtain the viral lysate, reinoculation, and incubation under continuous light at 15°C was repeated three times and yielded two cyanophage strains capable of producing cell lysis in cyanobacterial cultures P-101 and O-120. Transmission electron microscopy revealed an icosahedral capsid and contractile tail typical of members of the family *Myoviridae* (Fig. 1A through C). Analysis of the 16S rRNA gene of cultures P-101 and O-120 located the host in the *Synechococcus-Cyanobium* complex.

Viral DNA was extracted using precipitation with NaCl and polyethylene glycol 8000, followed by the use of a MasterPure complete DNA and RNA purification kit (Epicentre). DNA was fragmented in a Covaris sonicator and purified using magnetic beads (Axygen), and libraries were prepared using a NEBNext Ultra II kit for Illumina (New England Biolabs). DNA was sequenced using a MiSeq reagent kit v.2 (Illumina) at the Plateforme d’Analyses Génomiques de l’IBIS (Université Laval).

The total number of paired-end sequenced reads was 2,723,694, with an average length of 300 bp. All analyses were performed using default parameters, unless otherwise stated. Raw reads were filtered using Trimmomatic v.0.36 (2) to remove low-quality regions and Illumina adaptors. Reads were resampled using seqtk v.1.0-r31 (3) to reduce the assembly coverage to 100-fold (114,251 reads), and genomes were assembled *de novo* using SPAdes v.3.9.0 (4). We designed two primers, circ_B3 and circ_B23, to check the circularity of the genomes. PCRs were carried out in a 25-μl reaction mixture containing Platinum *Taq* polymerase (2 U; Thermo Fisher Scientific), PCR buffer with MgCl_2_, 0.2 mM deoxynucleoside triphosphates (dNTPs), and 0.4 μM each primer. Thermal cycling consisted of an initial step at 94°C for 2 min followed by 29 cycles of denaturation at 94°C for 15 s, hybridization at 50°C for 15 s, and elongation at 72°C for 1 min, with a final elongation step at 72°C for 5 min (7 min for circ_B23). Products were of the expected sizes (∼500 bp [B3] and 350 bp [B23]), confirming that the genome is circular and that the whole genome had been sequenced.

Genomes were annotated using RAST with the virus option (5). Percent similarity between the two viral strains was calculated using progressiveMauve v.2.4.0 (6). The two genomes were visualized using the R package circlize (7).

The genomes of B3 and B23 are circular and contain 244,930 bp and 243,633 bp, respectively, at the high end of the range for cyanomyoviruses (171 to 252 kb) (8). Nucleotide identity between the two genomes is 99.1%, and the G+C content is 34.8% for both genomes. No genomes with significant similarity to either B3 or B23 were found in the NCBI database. RAST identified 370 coding sequences (CDSs) in B3 and 365 CDSs in B23 (Fig. 1D), including 18 on the negative-sense strand for both novel viruses. Eighty percent of CDSs were annotated as hypothetical proteins, but only 20% could be assigned putative functions. Sequence divergence between the strains included the insertion/deletion of short repeat regions, as well as four contiguous CDSs present only in B3 (Fig. 1D).

Our strains possess only two known auxiliary metabolic genes (AMGs) (genes that may enhance the host metabolism during infection). They have the phosphate-starvation-induced gene *phoH* and the high-light-induced gene *hliP* but lack the widespread photosystem component gene *psbA.* Freshwater cyanophages generally have fewer photosynthetic AMGs than do marine cyanophages (9). Of the two other freshwater *Myoviridae* cyanophages with complete genome sequences of which we are aware, S-CRM01 (10) has *psbA* and Ma-LMM01 (11) does not.

The numbers of tRNAs in our genomes (14 for B23 and 16 for B3) are at the high end of the range for cyanomyophages. tRNAs may help viruses adapt to different host codon usage, which is an acute issue for *Synechococcus* phages or phages with broad host ranges (12). Broad host ranges would be adaptive in the subarctic region, where cyanobacterial populations are seasonally sparse.

### Data availability.

Raw reads have been deposited in the NCBI Sequence Read Archive (accession numbers SRR10273620, SRR10273621, and SRR10273622), and the assembled genomes have been deposited in GenBank (accession numbers MN695334 and MN695335).
